# Recurrent Pregnancy Loss: A Couple-Based Framework for Integrating Paternal Assessment

**DOI:** 10.3390/biomedicines14081866

**Published:** 2026-08-20

**Authors:** Nektaria Kritsotaki, Dimitrios Diamantidis, Nikoleta Koutlaki, Nikolaos Machairiotis, Panagiotis Tsikouras

**Affiliations:** 1 Department of Obstetrics and Gynecology, Democritus University of Thrace, 68100 Alexandroupolis, Greece; nkoutlak@med.duth.gr (N.K.); ptsikour@med.duth.gr (P.T.); 2 Department of Urology, Democritus University of Thrace, 68100 Alexandroupolis, Greece; 3 Third Department of Obstetrics and Gynecology, University General Hospital “ATTIKON”, Medical School, National and Kapodistrian University of Athens, 12462 Athens, Greece; nikolaosmachairiotis@gmail.com

**Keywords:** recurrent pregnancy loss, recurrent miscarriage, paternal factors, sperm DNA fragmentation, male infertility, semen analysis, sperm aneuploidy, paternal assessment

## Abstract

**Background/Objectives:** Recurrent pregnancy loss (RPL) has traditionally been investigated predominantly through maternal factors, while the clinical role of paternal assessment remains inconsistently defined. Current guidelines differ substantially regarding semen analysis, sperm DNA fragmentation (SDF), genetic testing, and referral for andrological evaluation. This review aimed to compare contemporary guideline recommendations, critically appraise the directness, prognostic value, and clinical utility of the supporting evidence, and classify paternal assessment strategies as routine, selective, or investigational. **Methods:** A structured narrative review was conducted using PubMed and Scopus searches through June 2026. International RPL, obstetric, reproductive medicine, and andrology guidelines were compared. Evidence from systematic reviews, meta-analyses, clinical studies, and clinically relevant molecular investigations was evaluated according to its directness to RPL populations, diagnostic and prognostic value, and evidence that test-guided interventions improve miscarriage or live-birth outcomes. **Results:** Routine paternal assessment should include age, reproductive and medical history, body weight, lifestyle, medication exposure, and relevant environmental or occupational risks. Conventional semen analysis is appropriate primarily when RPL coexists with infertility or suspected male reproductive disease. SDF is the most extensively studied advanced paternal biomarker and is frequently elevated in RPL cohorts, but findings vary by assay and comparator population, while prospective prediction of subsequent live birth and benefit from SDF-directed treatment remain unproven. Parental karyotyping has established counselling value but should be risk-stratified. Sperm aneuploidy testing, oxidative stress assays, seminal microbiome profiling, epigenetic biomarkers, and biomarker-directed interventions remain investigational. **Conclusions:** Paternal assessment in RPL should be couple-based, clinically targeted, and evidence-informed. Current evidence supports routine clinical evaluation, selective use of semen analysis, SDF testing, genetic assessment, and reproductive urology referral, and restriction of unvalidated biomarkers and treatments to research settings.

## 1. Introduction

Recurrent pregnancy loss (RPL) is a heterogeneous reproductive disorder with substantial clinical and psychological consequences. Its definition differs across professional organisations. The European Society of Human Reproduction and Embryology (ESHRE) considers that RPL can be diagnosed after two or more pregnancy losses. In contrast, the Royal College of Obstetricians and Gynaecologists (RCOG) defines recurrent miscarriage as three or more first-trimester pregnancy losses. The RCOG nevertheless encourages clinicians to consider extensive evaluation after two losses when these are suspected to be pathological rather than sporadic [1,2]. Despite extensive investigation, a substantial proportion of couples remain without an identified cause.

The evaluation of RPL has historically focused predominantly on the female partner. Established pathways assess uterine anatomy, antiphospholipid syndrome, endocrine and metabolic disorders, and parental chromosomal abnormalities. In contrast, paternal assessment has frequently been limited to general history or parental karyotyping, with limited agreement regarding conventional semen analysis, sperm DNA fragmentation (SDF), sperm aneuploidy, or specialist andrological evaluation [1,2,3]. This restricted role has increasingly been questioned because paternal age, obesity, metabolic health, sperm DNA integrity, sperm chromosomal abnormalities, and sperm epigenetic alterations have been associated with pregnancy loss or RPL in observational studies and meta-analyses [4,5,6,7,8,9]. Because reproductive outcomes arise from both partners, paternal associations should be interpreted within the phenotype of the couple, including maternal age, infertility status, reproductive history, and other shared or partner-specific factors that may confound apparent paternal effects.

The clinical significance of these associations remains uncertain. Higher SDF has been reported in several RPL case–control studies and meta-analyses, but its ability to predict the outcome of a subsequent pregnancy has not been established, and prospective evidence that SDF-directed treatment improves live birth is lacking [6,10,11]. Similar limitations apply to sperm aneuploidy, seminal microbiome profiling, and sperm epigenetic biomarkers. Evidence in these domains remains exploratory, with heterogeneous assays, small discovery cohorts, and limited prospective RPL data [7,9,12,13].

These uncertainties have resulted in marked divergence between current guidelines. Some RPL guidelines restrict advanced paternal testing to selected cases or research settings, whereas andrology guidelines adopt more permissive positions. ESHRE states that SDF testing may be considered for diagnostic purposes, whereas RCOG advises against its routine use outside research [1,2]. The amended American Urological Association and American Society for Reproductive Medicine (AUA/ASRM) male infertility guideline recommends male evaluation and SDF testing in couples with RPL as a moderate recommendation based on Grade C evidence [14,15]. In contrast, the European Association of Urology (EAU) Sexual and Reproductive Health Guideline gives a strong recommendation to perform SDF testing in couples with RPL following natural conception, failure of assisted reproductive technology, or unexplained male infertility [16]. This represents the most affirmative current guideline position, although the EAU also acknowledges heterogeneity in the evidence and the absence of studies directly demonstrating that SDF testing improves clinical management. These diagnostic recommendations should, therefore, not be interpreted as evidence that an abnormal SDF result should automatically alter treatment.

This review compares current guideline recommendations, critically evaluates the directness, prognostic value, and clinical actionability of the supporting evidence, and classifies paternal assessment domains as routine, selective, or investigational.

## 2. Materials and Methods

### 2.1. Study Design and Search Strategy

A structured narrative review of paternal assessment in recurrent pregnancy loss was conducted. The analysis included two components. First, current recommendations from reproductive medicine, obstetric, and andrology guidelines were compared to identify areas of agreement, divergence, and omission. Second, the evidence supporting these recommendations was assessed, with emphasis on whether it was derived directly from recurrent pregnancy loss populations or extrapolated from male infertility and assisted reproduction.

PubMed and Scopus were searched up to June 2026. Search terms included recurrent pregnancy loss, recurrent miscarriage, paternal factor, male factor, paternal age, semen analysis, sperm DNA fragmentation, sperm aneuploidy, sperm fluorescence in situ hybridization, parental karyotype, varicocele, oxidative stress, antioxidants, infection, microbiome, epigenetics, sperm methylation, male infertility, in vitro fertilization, and intracytoplasmic sperm injection. Reference lists of eligible guidelines, systematic reviews, and primary studies were also screened.

Because the review was designed as a structured narrative synthesis rather than a systematic review, the search was intended to provide comprehensive coverage of clinically relevant paternal domains rather than to generate a formally exhaustive study set for quantitative synthesis.

### 2.2. Eligibility Criteria

Eligible publications included formal clinical guidelines, committee opinions, systematic reviews, meta-analyses, randomized controlled trials, cohort studies, case–control studies, and clinically relevant molecular studies. Studies were included if they evaluated male partners of couples with recurrent pregnancy loss or assessed a paternal exposure, diagnostic test, or intervention relevant to pregnancy loss. Evidence from infertility or assisted reproduction populations was retained when it addressed tests or interventions commonly applied to recurrent pregnancy loss. Case reports, conference abstracts, nonclinical laboratory studies, and studies without extractable paternal data were excluded from the main synthesis.

Relevant publications were selected according to their clinical relevance to the predefined paternal domains and the directness of their evidence to RPL. For each included source, the authors extracted the population and clinical context, paternal exposure or investigation evaluated, principal reproductive or surrogate outcomes, major quantitative findings when reported, and key methodological limitations relevant to interpretation. Particular attention was given to whether evidence originated from RPL-specific populations or from broader miscarriage, infertility, or assisted reproduction cohorts. Study selection and data extraction were performed by the two lead authors, with any uncertainties resolved through discussion and consensus.

### 2.3. Guideline Selection

We included the most recent published versions of international reproductive medicine guidelines, national recurrent pregnancy loss or obstetric guidelines, and major andrology guidelines relevant to male evaluation. The included documents were issued by ESHRE [1], ASRM [17], RCOG [2], Royal Australian and New Zealand College of Obstetricians and Gynaecologists (RANZCOG) [18], Health Service Executive and Royal College of Physicians of Ireland (HSE/RCPI) [19], Society of Obstetricians and Gynaecologists of Canada (SOGC) [20], the guideline jointly issued by the German, Austrian, and Swiss Societies of Gynaecology and Obstetrics (DGGG, OEGGG, and SGGG) [3], AUA and ASRM [14,15] and EAU [16]. Andrology guidelines were included because the review examined whether investigations established in male infertility can be applied to recurrent pregnancy loss. The 2022 DGGG, OEGGG, and SGGG guideline was included as the most recent published version, although its formal validity expired in April 2025 and an update was in progress. When a guideline did not address a paternal domain, this was recorded as male partner not addressed.

### 2.4. Evidence Appraisal and Synthesis

Evidence was classified as direct recurrent pregnancy loss evidence, broader miscarriage evidence, infertility-derived evidence, or mechanistic evidence. Patient important outcomes included miscarriage, ongoing pregnancy, live birth, cumulative live birth, and time to live birth. Surrogate outcomes included semen parameters, sperm DNA fragmentation, oxidative stress, sperm aneuploidy, chromatin packaging, microbiome composition, and sperm methylation. Each paternal domain was assessed according to evidence directness, diagnostic value, prognostic value, clinical utility, and evidence that test-guided intervention improved miscarriage or live birth outcomes. Findings were classified as routine, selective, or investigational. Routine assessment was defined as broadly applicable evaluation supported by current clinical guidance and with established clinical relevance. Selective assessment referred to tests or referrals appropriate only when a specific clinical indication was present and for which diagnostic or counselling value exceeded evidence supporting universal use. Investigational assessment included tests or interventions lacking standardized thresholds, prospective prognostic validation, or evidence that test-guided management improves reproductive outcomes. Evidence was synthesized narratively by clinical domain, with attention to assay heterogeneity, control group selection, confounding, and the distinction between association and prospective prediction.

Given the broad and heterogeneous evidence base, which included clinical guidelines, evidence syntheses, randomized and observational studies, and molecular investigations, no single study-level risk-of-bias instrument was applicable across all included evidence. Methodological limitations were, therefore, evaluated and reported within each clinical domain, with particular emphasis on population directness, comparator selection, heterogeneity, confounding, prospective validation, and the use of surrogate rather than patient-important reproductive outcomes.

A de novo GRADE assessment was not performed because this structured narrative review included heterogeneous evidence sources and was not designed as a formal evidence-grading exercise. Evidence strength was instead interpreted according to study design, directness to RPL populations, consistency, prospective validation, and clinical utility.

## 3. Why Guidelines Disagree

### 3.1. Guideline Spectrum

Current recommendations for paternal assessment in recurrent pregnancy loss (RPL) vary most clearly in relation to sperm DNA fragmentation (SDF) testing. ESHRE recommends assessment of paternal age, smoking, alcohol consumption, exercise, and body weight. SDF testing may be considered for diagnostic purposes, although this is a conditional recommendation and no specific treatment pathway is proposed after an abnormal result [1]. The 2026 ASRM committee opinion adopts a selective approach. Standard semen parameters are not considered predictive of RPL, while SDF testing and reproductive urology evaluation are recommended in selected cases of unexplained RPL or concurrent infertility [17].

The Royal College of Obstetricians and Gynaecologists (RCOG) recognises paternal age as a risk factor but advises against routine SDF testing outside research. The guideline acknowledges the reported association between increased SDF and recurrent miscarriage but considers the evidence insufficient to support routine clinical use or a defined treatment strategy [2]. The HSE/RCPI similarly recommend assessment of paternal age, smoking, alcohol consumption, medication use, exercise, and body weight while advising against routine SDF screening outside research. They also conclude that current evidence does not support a specific treatment for male factors in recurrent miscarriage [19].

SOGC takes a more permissive position. Male history and examination should assess age, body mass index (BMI), smoking, alcohol use, heat exposure, toxins, infection, and palpable varicocele. SDF testing can be offered when no other abnormality has been identified, although evidence supporting lifestyle intervention, antioxidant treatment, or the use of testicular sperm remains limited [20]. RANZCOG also permits SDF testing on a case-by-case basis for diagnostic purposes. The guideline recognises paternal age, lifestyle factors, and potentially modifiable male conditions but emphasises the limited evidence that treatment of these factors improves RPL outcomes [18].

The guideline DGGG, OEGGG, and SGGG addresses paternal age and couple-based cytogenetic assessment but does not provide an operational recommendation for SDF testing, conventional semen analysis, or a broader male investigation pathway [3]. Parental karyotyping is considered within the genetic evaluation of the couple rather than as part of a structured andrological assessment.

The andrology guidelines occupy the more supportive end of the spectrum. The amended AUA/ASRM male infertility guideline recommends evaluation of the male partner in couples with RPL and includes SDF testing as a moderate recommendation supported by Grade C evidence. Paternal karyotyping is recommended as expert opinion, while sperm aneuploidy testing may be considered. The guideline also states that controlled evidence is lacking for testicular sperm retrieval, antioxidants, donor sperm, varicocele repair, or frequent ejaculation as interventions to reduce RPL [14,15].

The EAU guideline recommends performing SDF testing in couples with RPL. This recommendation is graded as strong, although RPL is considered together with unexplained infertility and failure of assisted reproductive technology (ART). The guideline does not recommend routine reactive oxygen species (ROS) testing and considers the use of testicular sperm in men with high SDF experimental [16].

The recommendations, therefore, form a continuum. At one end, the EAU recommends SDF testing, while the AUA/ASRM guideline recommends male evaluation with SDF assessment based on moderate, Grade C evidence. The ASRM committee opinion, SOGC, ESHRE, and RANZCOG support testing only in selected clinical circumstances. RCOG and HSE/RCPI advise against routine use outside research, while DGGG, OEGGG, and SGGG do not provide a specific recommendation. Table 1 compares the recommendations across the current guidelines.

### 3.2. Explanation of Divergence

Part of this divergence reflects the clinical perspective from which the evidence is evaluated. Andrology guidelines assess RPL within the wider context of male reproductive disease. Identification of an abnormal sperm biomarker or a potentially treatable male condition may, therefore, be considered sufficient to justify further evaluation. Guidelines focused primarily on RPL place greater emphasis on whether a test predicts the outcome of a subsequent pregnancy and whether management based on the result improves live birth.

The recommendations also apply different thresholds to diagnostic evidence. Case–control studies reporting higher SDF in male partners of couples with RPL establish an association with previous pregnancy losses. They do not necessarily establish a prospective prediction of another miscarriage. Guidelines that regard case–control evidence as sufficient to support diagnostic use are more favourable toward testing, whereas those requiring independent prognostic validation remain selective or restrictive.

Another source of disagreement is the importance assigned to treatment utility. An abnormal SDF result may identify a biological abnormality, but current evidence does not establish that treatment selected according to this result reduces miscarriage or improves live birth. Some panels, therefore, consider routine testing premature. Others support selective testing for diagnostic clarification, counselling, or identification of coexisting male reproductive disease despite the absence of a validated outcome-directed treatment pathway.

Evidence transferability is also interpreted differently. Much of the literature on SDF-directed interventions, oxidative stress, varicocele treatment, antioxidants, and testicular sperm originates from infertility or assisted reproductive technology populations rather than from RPL-specific cohorts. Andrology guidelines are more willing to integrate evidence across related reproductive indications, whereas RPL-focused guidelines generally require more direct evidence from the target population.

The low physical burden of SDF testing may further lower the threshold for selective use. However, procedural simplicity does not establish clinical utility. An abnormal result may initiate a cascade of repeated investigations, specialist consultations, unproven interventions, additional costs, or escalation to assisted reproductive technology without a clearly validated decision pathway.

### 3.3. Core Interpretation

The disagreement does not mainly concern whether paternal abnormalities are present in recurrent pregnancy loss. It concerns whether their detection provides sufficiently reliable and actionable information to justify routine testing. Association with previous loss, prediction of future pregnancy outcome, and benefit from test-directed treatment are distinct evidentiary levels. Current evidence is strongest for association, less consistent for prognosis, and insufficient for treatment-guided improvement in live birth. The position adopted by each guideline depends largely on which of these levels it considers necessary for clinical implementation.

## 4. Clinical Evidence by Paternal Domain

### 4.1. Routine Paternal Assessment

Paternal assessment should begin with reproductive and medical history, age, lifestyle, medication exposure, body weight, and symptoms or signs of male reproductive disease. This approach has low cost and may identify factors relevant to general health, fertility, and pregnancy outcome without requiring advanced laboratory testing.

The association between paternal age and miscarriage is supported by a systematic review restricted to studies that adjusted for maternal age. Compared with men aged 25 to 29 years, the pooled odds of miscarriage increased at 40 to 44 years and at 45 years or older, with odds ratios of 1.23 and 1.43, respectively. The association was stronger for first-trimester miscarriage among men aged at least 45 years [4]. However, these estimates relate mainly to miscarriage in general rather than exclusively to RPL. In a cohort of 506 couples evaluated after RPL, paternal age was not independently associated with subsequent live birth after adjustment for other prognostic factors [8]. Paternal age is, therefore, relevant to counselling but should not be used as an isolated indication for advanced testing.

Paternal metabolic health may also contribute to pregnancy outcomes. In a large insurance claims cohort, increasing paternal metabolic syndrome burden was associated with a modest increase in spontaneous pregnancy loss after adjustment for maternal factors. Relative risks ranged from 1.10 with one metabolic component to 1.19 with three or more components [5]. As with the paternal age evidence, this study evaluated pregnancy loss in a broad population and was not restricted to couples with RPL. In the RPL-specific cohort reported by Peuranpää et al., paternal obesity was independently associated with a lower probability of live birth, with an age-adjusted hazard ratio of 0.67 [8]. Weight reduction can improve selected semen parameters in obese men, but the available intervention studies were conducted in infertility populations and did not assess recurrent miscarriage or live birth after RPL [21].

These findings support routine assessment and optimisation of paternal health, particularly smoking, alcohol use, obesity, medication exposure, recreational drug use, heat exposure, and chronic disease. The rationale is strongest for general preconception health and identification of established male reproductive disease. It remains uncertain whether modification of these factors after RPL directly reduces subsequent miscarriage. Reviews of miscarriage epidemiology and paternal contributions similarly distinguish biological relevance from unproven RPL-specific treatment benefit [22,23,24,25,26,27].

### 4.2. Conventional Semen Analysis

Conventional semen analysis assesses fertility potential but has limited ability to explain pregnancy loss. Case–control studies and meta-analyses have reported reduced sperm motility or fewer normal forms in men from RPL couples. In a small study of 22 RPL partners and 20 fertile controls, total and progressive motility were lower, and abnormal morphology was more frequent in the RPL group [28]. A meta-analysis of studies conducted in Chinese populations also reported poorer motility and morphology among male partners of women with recurrent spontaneous abortion, although between-study heterogeneity was substantial [6]. In a broader meta-analysis focused primarily on SDF, secondary analyses suggested lower total and progressive motility in men from RPL couples, while sperm concentration and total sperm count were not significantly different [29].

More recent evidence has not consistently reproduced clinically meaningful differences in conventional semen parameters. In a comparative study of 1485 men, including 634 male partners from RPL couples and 851 controls, neither conventional semen parameters nor SDF were independently associated with RPL [30]. Other direct RPL studies and meta-analyses have reported normal or overlapping semen parameters despite abnormalities in sperm DNA integrity, aneuploidy, chromatin packaging, or related molecular markers [11,31,32]. The magnitude of reported differences also depends strongly on control-group selection. Comparisons with recently proven fertile men generally produce larger contrasts than comparisons with men attending fertility services.

Conventional semen parameters primarily describe the probability of conception and do not directly measure the paternal genomic contribution to embryo development. A normal semen analysis does not exclude increased SDF, sperm aneuploidy, altered protamination, or epigenetic abnormalities [28,33,34]. Conversely, a minor reduction in motility or morphology does not establish causation for recurrent loss.

Routine semen analysis solely to predict another miscarriage is, therefore, not supported. It remains appropriate when RPL coexists with delayed conception, abnormal male reproductive history, previous testicular or genital disease, sexual dysfunction, or clinical suspicion of male infertility. In this setting, its purpose is to identify concomitant infertility and guide standard andrological assessment rather than to provide an RPL-specific prognostic test [35,36].

### 4.3. Sperm DNA Fragmentation

Sperm DNA fragmentation is the most extensively studied advanced paternal test in RPL. Several systematic reviews and meta-analyses have found higher mean SDF in male partners of women with RPL than in fertile controls. McQueen et al. reported a mean difference of approximately 11.9 percentage points, while Tan et al. reported a difference of approximately 12.0 percentage points in idiopathic RPL [6,10]. Subsequent meta-analyses reached similar conclusions, although effect estimates varied according to RPL definition, assay, threshold, control population, and study quality [29,37].

Primary studies have generally supported an association, although the magnitude and consistency of the effect vary. Elevated SDF has been reported using terminal deoxynucleotidyl transferase dUTP nick end labelling, the sperm chromatin structure assay, sperm chromatin dispersion, and Comet-based methods [11,28,38]. A meta-analysis by Yuan et al. also reported higher SDF in RPL despite overlapping conventional semen parameters [31]. In a multicentre case–control study, median SDF was higher in idiopathic RPL than in fertile controls. After multivariable adjustment, however, the association was modest, with an adjusted odds ratio of 1.13 per unit increase [11].

More recent assay-specific evidence illustrates the heterogeneity of the field. Drakeley et al. compared 100 male partners from couples with unexplained recurrent first-trimester miscarriage with 81 proven-fertile sperm donors. Both global and double-stranded sperm DNA damage were higher in the RPL group, but double-stranded damage measured using the neutral Comet assay showed substantially stronger discrimination, with areas under the curve of 0.876 and 0.909 for the two reported double-stranded damage metrics [38]. Many affected men had normal conventional semen parameters. These findings support the possibility that specific forms of DNA damage may be more informative than a composite SDF measure, but the study used an unmatched, substantially younger donor control group, assay-specific thresholds, and did not assess future pregnancy outcomes.

Not all direct studies have been positive. Esquerré-Lamare et al. found increased sperm aneuploidy but no significant increase in SDF measured by either the sperm chromatin structure assay or terminal deoxynucleotidyl transferase dUTP nick end labelling [32]. In a comparative study of 1485 men, including 634 RPL partners and 851 fertility-clinic controls, neither continuous DNA fragmentation index nor a threshold above 30% was independently associated with RPL after adjustment [30]. Rasmussen et al. similarly found no increase in SDF among men from couples with unexplained RPL. SDF was lower in the RPL group than in proven-fertile controls in that study, and seminal oxidative stress did not differ significantly between groups [39].

The contrast between Yao et al. and Drakeley et al. is particularly informative. Yao et al. used the sperm chromatin structure assay and found no independent association, whereas Drakeley et al. reported a stronger signal for double-stranded damage measured by neutral Comet testing [30,38]. These findings suggest that apparently conflicting results may partly reflect differences in the biological lesions measured, assay methodology, threshold selection, and comparator population rather than a simple distinction between positive and negative studies.

The principal limitation is that most studies compare previous RPL status with fertile or fertility-clinic control status. This establishes diagnostic association but not prediction of the next pregnancy. In the prospective RPL cohort reported by Peuranpää et al., live birth occurred in 71.1% of couples with normal DFI and 63.3% of those with high DFI, a non-significant difference [8]. Cross-sectional correlations between SDF and the number of previous losses likewise cannot be interpreted as prospective prognostic evidence.

Evidence linking sperm DNA damage to miscarriage after assisted reproductive technology is more extensive but less directly transferable. Meta-analyses of in vitro fertilisation and intracytoplasmic sperm injection populations have associated elevated SDF with increased miscarriage, including risk estimates of approximately twofold in some analyses [40,41]. These populations differ from naturally conceiving RPL couples in patient selection, sperm processing, embryo selection, and treatment pathway. The findings support biological plausibility but do not establish that SDF testing improves RPL management.

Assay heterogeneity remains clinically important. Terminal deoxynucleotidyl transferase dUTP nick end labelling detects DNA strand breaks, the sperm chromatin structure assay assesses susceptibility to acid-induced denaturation, sperm chromatin dispersion evaluates halo formation, and Comet assays quantify migration of fragmented DNA. These methods are not interchangeable and use different thresholds [42,43,44]. Abstinence duration, infection, fever, oxidative stress, laboratory processing, and intraindividual variation may further affect results.

SDF, therefore, has the strongest evidence among advanced paternal biomarkers for an association with RPL, but its independent prognostic value and treatment utility remain uncertain. Testing may be considered selectively in unexplained RPL, particularly when RPL coexists with infertility, abnormal semen parameters, or other clinical features suggesting male reproductive disease. In isolated RPL without an additional male-factor indication, the clinical utility of SDF testing remains less certain.

### 4.4. Genetic Assessment

Parental karyotyping differs from sperm biomarker testing because detection of a balanced chromosomal rearrangement has established explanatory and counselling implications. A contemporary meta-analysis estimated that parental chromosomal abnormalities are present in approximately 5% of RPL couples, although this estimate combined maternal and paternal carriers and showed substantial heterogeneity [45]. Couples with an abnormal parental karyotype had a lower live-birth rate in the first subsequent pregnancy than couples with normal karyotypes, with the reduction driven mainly by reciprocal and Robertsonian translocations [46].

The diagnostic yield depends strongly on prior risk. In a retrospective study of 213 RPL couples, pathogenic numerical or structural abnormalities were detected in 3.75% of individuals. After exclusion of couples already selected because of an abnormal pregnancy-tissue or prenatal cytogenetic result, the yield fell to 0.94% [47]. This supports targeted testing after an unbalanced pregnancy-tissue result, previous offspring with congenital anomalies, suggestive family history, or unavailable tissue analysis rather than universal testing of all male partners. Robertsonian translocation rob(13;14) is enriched in RPL cohorts, but the evidence derives from heterogeneous cytogenetic series and narrative synthesis rather than prospective screening studies [48].

Identification of a balanced rearrangement permits genetic counselling, prenatal diagnosis, and discussion of preimplantation genetic testing for structural rearrangements (PGT-SR). It does not necessarily imply that assisted reproduction improves cumulative live birth. In the systematic review by Li et al., couples with abnormal parental karyotypes had a first-pregnancy live-birth rate of 58.5%, compared with 71.9% among non-carriers. The association was stronger among couples carrying reciprocal translocations, with live-birth rates of 49.5% versus 75.2%, and among Robertsonian translocation carriers, with rates of 58.1% versus 75.2% [46]. However, cumulative live birth did not differ significantly between carriers and non-carriers. Evidence comparing PGT-SR with expectant management was limited to two small non-randomized studies and did not demonstrate superiority of PGT-SR for cumulative live birth, although miscarriage rates were lower after PGT-SR [46].

Sperm aneuploidy represents a separate genomic domain. Conventional blood karyotyping cannot detect de novo meiotic errors confined to spermatozoa. Small case–control studies have reported higher sperm aneuploidy in RPL, including abnormalities involving chromosomes X, Y, 13, 18, and 21 [28,32,33]. A meta-analysis of nine quantitative studies involving 326 male partners from RPL couples and 124 fertile controls found higher total sperm aneuploidy in the RPL group, with a standardized mean difference of 1.07. However, only three studies contributed to the primary pooled analysis, and heterogeneity was substantial [7]. Chromosome panels, fluorescence in situ hybridisation methods, scoring criteria, thresholds, and RPL definitions differed markedly between studies.

Sperm fluorescence in situ hybridisation has not been validated as a predictor of subsequent miscarriage or live birth. No evidence shows that fluorescence in situ hybridisation-guided PGT-SR, donor sperm, sperm selection, or modification of assisted reproductive technology improves outcomes in RPL. It should therefore remain investigational rather than form part of routine paternal assessment.

### 4.5. Varicocele, Oxidative Stress, and Male-Directed Treatment

Oxidative stress is a biologically plausible mechanism linking obesity, smoking, heat exposure, infection, varicocele, and systemic disease with sperm DNA damage. The RPL-specific literature, however, remains limited and conflicting. Davies et al. reviewed the mechanistic and clinical evidence linking seminal oxidative stress with RPL but identified substantial methodological heterogeneity and concluded that the value of antioxidant treatment remains uncertain [49]. In the direct case–control study by Rasmussen et al., normalized seminal oxidation-reduction potential did not differ significantly between men from couples with unexplained RPL, unexplained infertility, and proven fertility. SDF was also not elevated in the RPL group [39]. These findings do not support routine oxidative stress testing in RPL.

Varicocelectomy can reduce SDF in infertile men with clinical varicocele. A meta-analysis of 19 studies involving 1070 patients reported a mean postoperative reduction of 7.23 percentage points, although heterogeneity was substantial [50]. Antioxidant supplementation following varicocele treatment may also improve selected semen or oxidative stress measures, but the available studies involve infertility populations and predominantly report surrogate outcomes [51]. These data do not demonstrate fewer miscarriages or more live births in RPL. Varicocele assessment and repair should therefore follow established infertility and andrology indications rather than an assumed RPL-specific benefit.

Antioxidant research illustrates the distinction between biomarker change and clinically relevant treatment benefit. Habibi et al. conducted a post hoc analysis of 37 men with elevated sperm DNA damage whose partners had RPL. Alpha-lipoic acid was associated with within-group improvements in SCSA-derived DNA damage, total motility, lipid peroxidation, protamine deficiency, and retained histones. However, treatment effects were not consistently supported by between-group comparisons, and oxidative stress findings were mixed. Pregnancy and live-birth differences were not statistically significant, and the analysis was underpowered for reproductive outcomes [52].

A Cochrane review of antioxidants for male subfertility identified low or very low certainty evidence, substantial methodological limitations, and no direct basis for treatment of RPL couples [53]. The multicentre SUMMER randomized trial provides stronger indirect evidence. Among 1171 men seeking fertility care, six months of combined antioxidant supplementation did not improve ongoing pregnancy, first-trimester pregnancy loss, semen parameters, or SDF. During the prespecified period of expected treatment effect between four and six months, ongoing pregnancy was lower in the antioxidant group than in the placebo group [54]. Although this was not an RPL population, the findings argue against extrapolating favourable biomarker changes into routine empirical antioxidant treatment.

Testicular sperm retrieval, shortened abstinence, donor sperm, advanced sperm-selection methods, and empirical escalation to assisted reproductive technology have not been validated as RPL-directed interventions. Lower SDF has been reported in testicular than in ejaculated sperm in selected infertile men, but this evidence derives mainly from assisted reproduction populations and does not demonstrate improved cumulative live birth in RPL [42]. Current guideline discussions similarly acknowledge the absence of controlled RPL-specific evidence supporting these interventions solely on the basis of elevated SDF [14,15,16] (AUA and ASRM, 2024; EAU, 2026). Male-directed treatment should therefore address established reproductive disease and standard infertility indications. Intervention undertaken solely to correct an abnormal paternal biomarker in an RPL couple remains investigational.

### 4.6. Infection, Microbiome, and Emerging Biomarkers

Evidence supporting routine infection screening in asymptomatic male partners from RPL couples is limited. A meta-analysis found that seminal human papillomavirus (HPV) infection was associated with higher SDF and an increased risk of miscarriage, with an odds ratio of 5.13 [55]. However, the included studies involved heterogeneous infertility and assisted reproductive technology populations, used different HPV detection methods, and did not establish that viral clearance or treatment reduces miscarriage in RPL. Semen culture and targeted infection testing remain appropriate when symptoms, pyospermia, leukocytospermia, or other clinical findings suggest genital tract infection, but current RPL evidence does not support indiscriminate screening or empirical antimicrobial treatment in asymptomatic men.

The first substantial study specifically evaluating the seminal microbiome in an RPL subgroup did not identify a distinct RPL-related microbial profile. The cohort included 223 men, of whom 46 were partners in couples with RPL, 58 had male-factor infertility, 56 were partners in couples with unexplained infertility, and 63 had proven fertility. Bacterial richness, alpha diversity, bacterial load, and overall community composition did not differ significantly between the clinical groups. Global microbiome characteristics were also not associated with semen quality, SDF, or seminal reactive oxygen species. The only notable taxon-level association involved unidentified Flavobacterium and abnormal semen analysis rather than RPL itself [13]. These findings argue against routine seminal microbiome profiling in RPL. Microbiome-directed treatment remains investigational.

Sperm epigenetic studies have identified potentially relevant molecular differences. In a case–control study of 112 couples with idiopathic RPL and 106 proven-fertile couples, Khambata et al. reported reduced global sperm DNA methylation, hypomethylation at specific CpG sites within LINE-1, IGF2-H19 DMR, and IG-DMR, and hypermethylation at maternally imprinted loci including MEST, ZAC, KvDMR, PEG3, and PEG10 [9]. These findings support a possible paternal epigenetic contribution but remain associative and were not linked to subsequent pregnancy outcomes.

A subsequent diagnostic-development study evaluated whether these imprinted-gene abnormalities could distinguish male partners from RPL couples from fertile controls. PEG3 methylation showed an area under the curve of 0.88, with 70% sensitivity and 90.4% specificity at the reported threshold [12]. Although these findings demonstrate case–control discrimination, they do not establish prospective prognostic value, external validity, or clinical actionability.

Genome-wide sperm methylation analysis has provided an additional independent signal. Wei et al. identified differentially methylated CpG sites and regions in men from couples with unexplained RPL and reported H19 hypermethylation on targeted validation. The reported H19 measures showed areas under the curve of 0.784 and 0.813, with sensitivity and specificity of approximately 80% [56]. The direction of the H19-related methylation abnormality was not fully concordant with the hypomethylation reported by Khambata et al., possibly reflecting differences in CpG sites, genomic regions, assays, and populations. This inconsistency, together with the absence of external validation and pregnancy-outcome data, limits clinical interpretation.

Evidence concerning sperm LINE-1 methylation outside RPL further illustrates the need to distinguish mechanistic background from direct clinical evidence. Crafa et al. evaluated associations between sperm LINE-1 methylation, semen parameters, and paternal age, but did not include an RPL cohort or miscarriage outcomes [57]. The study therefore provides general epigenetic context rather than direct evidence for paternal assessment in RPL.

Other chromatin-related abnormalities have also been reported. Rogenhofer et al. found altered protamine-1 and protamine-2 messenger RNA content and an abnormal protamine transcript ratio in sperm from men whose partners had unexplained recurrent miscarriage [58]. Abnormal chromatin packaging has also been described using research assays in small RPL cohorts [28]. These findings support biological plausibility but lack standardized thresholds, independent validation, and evidence of prognostic or treatment utility.

Proteomic, transcriptomic, methylomic, chromatin, and microbiome approaches therefore remain research tools. Narrative reviews have catalogued these emerging paternal mechanisms, but none has established routine clinical utility or a validated management pathway [26,34,59,60].

Emerging integrative approaches increasingly seek to combine genomic, epigenomic, transcriptomic, proteomic, metabolomic, microbiome, and single-cell data rather than evaluate individual biomarkers in isolation. In paternal RPL, however, such evidence remains preliminary. Recent work has integrated sperm gene-expression and DNA-methylation profiles with single-cell transcriptomic data and machine-learning models to identify candidate paternal imprinted-gene signatures associated with idiopathic RPL [61]. More broadly, AI and systems-biology approaches are increasingly being explored in RPL and male infertility for biomarker discovery, multidimensional risk prediction, and integration of molecular with clinical data [62,63]. Nevertheless, current studies remain limited by small or retrospective datasets, heterogeneous phenotyping, limited external validation, and the absence of evidence that multi-omics- or AI-guided paternal assessment improves subsequent live birth. Future work should therefore prioritize prospective multicentre validation and integrated couple-level models combining paternal and maternal molecular and clinical data.

Overall, routine paternal history and clinical assessment are justified. Conventional semen analysis is selective and is primarily relevant when infertility or suspected male reproductive disease coexists. SDF may provide explanatory information in selected couples, while parental karyotyping should be guided by individual genetic risk. Sperm fluorescence in situ hybridisation, oxidative stress assays, seminal microbiome profiling, epigenetic panels, and biomarker-directed treatment remain investigational. Across these domains, evidence of association is substantially stronger than evidence of prospective prognosis or treatment-related improvement in live birth. Table 2 summarizes the evidence base, principal findings, prognostic or treatment utility, and proposed clinical status of the main paternal assessment domains in RPL.

## 5. Clinical Implications and Evidence-Informed Stratification

Paternal assessment in recurrent pregnancy loss should be integrated into evaluation of the couple and applied according to clinical context, evidence directness, and the likelihood that a finding will change counselling or management. The available evidence supports routine low-burden clinical assessment, selective use of established male reproductive tests, and restriction of unvalidated biomarkers to research settings.

Initial assessment should include paternal age, reproductive and medical history, body weight, smoking, alcohol consumption, recreational drug use, medication exposure, occupational or environmental risks, heat exposure, and symptoms suggestive of male reproductive disease [1,18,19,20]. Counselling should address modifiable health factors as part of general preconception care. Focused male examination is appropriate when the history, fertility status, or symptoms raise suspicion of varicocele, testicular disease, endocrine dysfunction, genital tract pathology, or another andrological condition. Paternal age or obesity alone should not be used as an automatic indication for advanced laboratory testing, because their value for predicting the outcome of the next pregnancy remains limited [4,5,8].

Conventional semen analysis should not be requested solely to predict recurrent miscarriage. It is appropriate when RPL coexists with delayed conception, sexual dysfunction, abnormal male reproductive history, previous testicular or genital disease, abnormal examination, or suspected endocrine or male-factor infertility. In this setting, semen analysis identifies concomitant infertility and guides standard andrological evaluation rather than providing an RPL-specific prognostic test [17,29,30,36]. Normal conventional semen parameters do not exclude SDF, sperm aneuploidy, chromatin abnormalities, or epigenetic alterations.

SDF testing may be considered in selected couples with unexplained RPL, particularly when concurrent infertility is present or reproductive urology assessment is otherwise clinically indicated [1,14,15,17]. Repeated assisted reproductive technology failure is included within the broader European Association of Urology testing framework, but represents an adjacent infertility indication rather than direct RPL evidence [16]. Meta-analyses and several case–control studies report higher SDF in RPL, although findings vary by assay and comparator population [6,10,11]. Conversely, Yao et al. found no independent association after adjustment, and prospective evidence has not established SDF as a predictor of subsequent live birth [8,30]. An abnormal result should therefore be presented as a possible explanatory finding rather than a validated prognostic marker or an independent indication for treatment.

Genetic assessment should be risk-stratified. Pregnancy-tissue analysis, when available, provides the most direct information regarding the chromosomal basis of an individual loss. Parental karyotyping is most informative after identification of an unbalanced structural abnormality in pregnancy tissue, when pregnancy-tissue analysis is unavailable, or when family or reproductive history raises suspicion of a balanced rearrangement [46,47]. Detection of a balanced rearrangement warrants genetic counselling and discussion of natural conception, prenatal diagnosis, and preimplantation genetic testing for structural rearrangements. It does not establish that assisted reproduction improves cumulative live birth. Sperm fluorescence in situ hybridisation should not form part of routine evaluation because prognostic performance and test-directed management have not been validated [7].

Reproductive urology referral should be considered when RPL coexists with infertility, abnormal semen parameters, palpable varicocele, suspected endocrine or testicular disease, sexual dysfunction, or a significant male reproductive history. Referral may also be appropriate in selected unexplained RPL cases when advanced paternal testing is being considered. This pathway is supported more explicitly by the 2026 ASRM committee opinion and the AUA/ASRM male infertility guideline than by most obstetric RPL guidelines [14,15,17].

Treatment should address established male reproductive disease and standard infertility indications. Varicocele repair should follow accepted andrological criteria and should not be undertaken solely to reduce miscarriage risk. Empirical antioxidants should not be prescribed solely because SDF or another sperm biomarker is abnormal, because biomarker improvement has not translated into established reproductive benefit [52,54]. Testicular sperm retrieval, shortened abstinence, advanced sperm-selection methods, donor sperm, and escalation to assisted reproductive technology should not be adopted as routine RPL-directed interventions in the absence of an independent infertility indication [14,15,16].

Targeted infection testing is appropriate when symptoms, pyospermia, leukocytospermia, or other findings suggest genital tract infection. Empirical antimicrobial treatment should not be used in asymptomatic men without a documented infectious indication. Seminal human papillomavirus has been associated with miscarriage in mixed infertility and assisted reproduction populations, but no RPL-specific evidence demonstrates that viral clearance reduces subsequent loss [55]. Seminal microbiome profiling should remain investigational because no reproducible RPL-specific microbial signature or validated microbiome-directed treatment pathway has been identified [13].

In clinical practice, paternal history, preconception counselling, and clinically indicated examination constitute the routine tier. Semen analysis, SDF testing, parental karyotyping, and reproductive urology referral belong to the selective tier and should be used only when the clinical context provides a defined indication. Sperm aneuploidy testing, oxidative stress assays, seminal microbiome profiling, epigenetic or other omics panels, and biomarker-directed interventions remain investigational because validated thresholds, prospective prognostic performance, and effective test-guided management pathways have not been established.

**Figure 1:**
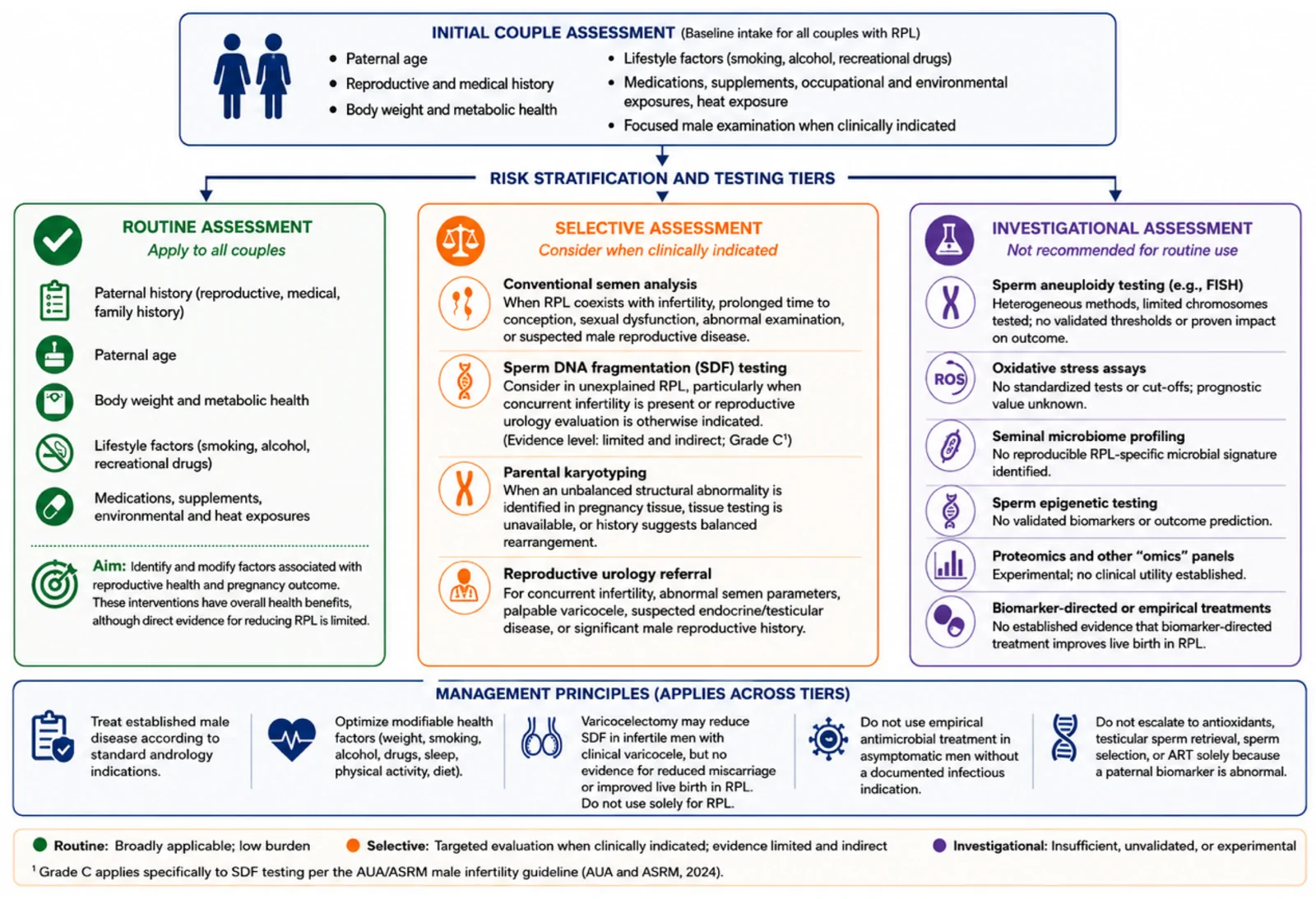
Evidence-informed paternal assessment framework in recurrent pregnancy loss (RPL). The Grade C designation shown in the figure refers specifically to SDF testing according to the AUA/ASRM male infertility guideline [14,15]. Abbreviations: ART, assisted reproductive technology; ASRM, American Society for Reproductive Medicine; RPL, recurrent pregnancy loss; SDF, sperm DNA fragmentation.

Practical implementation should also consider test availability, resource use, and the likelihood that results will alter management. SDF testing should remain selective, given its uncertain prognostic and treatment utility [8,30], while epigenetic and microbiome assays lack sufficient validation for routine clinical use [13,56]. Access to advanced testing and reproductive urology expertise may vary across healthcare settings, and broader implementation should therefore consider feasibility and cost-effectiveness. A tiered approach prioritizing low-burden clinical assessment, selective testing, and restriction of investigational assays to research settings appears most appropriate. Figure 1 summarizes the proposed evidence-informed framework for paternal assessment in recurrent pregnancy loss.

## 6. Evidence Gaps

The principal limitation of the paternal RPL literature is that most studies evaluate association with a previous history of pregnancy loss rather than prognosis in a subsequent pregnancy. Many studies compare men from RPL couples with fertile or infertility controls and report differences in semen characteristics, SDF, sperm aneuploidy, oxidative stress, or molecular biomarkers. Far fewer studies assess whether these findings independently predict miscarriage or live birth after RPL evaluation. The prospective RPL cohort reported by Peuranpää et al. found no significant association between SDF and subsequent live birth, illustrating the distinction between retrospective group discrimination and clinically relevant prognostic performance [8].

Substantial heterogeneity also affects the definition and selection of RPL populations. Studies differ in the required number of losses, inclusion of biochemical pregnancies, gestational-age limits, whether losses were consecutive, and whether couples with recognised maternal or genetic causes were excluded. Control groups range from proven-fertile sperm donors to men undergoing fertility evaluation, producing markedly different baseline risk profiles [30,38]. These differences limit comparability between studies and may partly explain why some cohorts identify substantial paternal abnormalities while others report weak or absent associations.

SDF research is additionally limited by variation in assay methodology, laboratory processing, abstinence periods, thresholds, and the biological lesions measured by each test. The sperm chromatin structure assay, terminal deoxynucleotidyl transferase dUTP nick end labelling, sperm chromatin dispersion, alkaline Comet, and neutral Comet assays are not interchangeable, and a threshold derived from one platform cannot be assumed to have equivalent meaning with another [6,10,29]. The contrast between the negative sperm chromatin structure assay findings of Yao et al. and the stronger discrimination reported using double-stranded Comet testing by Drakeley et al. demonstrates the extent to which assay selection and comparator choice can influence results [30,38]. Standardised pre-analytical procedures, assay-specific reference ranges, and externally validated RPL thresholds are therefore required before SDF can function as a reproducible prognostic test.

Paternal effects are also difficult to separate from maternal and couple-level confounding. Paternal age, obesity, metabolic disease, smoking, environmental exposures, infertility duration, and use of assisted reproductive technology frequently coexist with maternal age and other recognised determinants of pregnancy outcome [4,5,8]. Future studies should recruit and analyse couples rather than treating paternal variables in isolation. Models should adjust for maternal age, previous live birth, number and gestational timing of losses, maternal comorbidity, conception method, pregnancy-tissue findings, and concurrent infertility. Without this approach, an observed paternal association may represent shared exposure, couple-level subfertility, or residual confounding rather than an independent causal effect.

The evidence base is particularly weak for clinical utility. An abnormal paternal biomarker may identify a biological difference without improving prediction, counselling, or treatment selection. SDF, sperm fluorescence in situ hybridisation, oxidative stress testing, seminal microbiome analysis, and sperm epigenetic markers have not undergone sufficient prospective validation against subsequent live birth or miscarriage in independent RPL cohorts [7,9,12,13,56]. Reported diagnostic discrimination in case–control studies should not be interpreted as evidence that a test improves clinical decision-making.

Test-guided intervention evidence is almost absent. Recommendations concerning lifestyle optimisation, antioxidants, varicocele treatment, sperm selection, testicular sperm retrieval, frequent ejaculation, or escalation to assisted reproductive technology are commonly extrapolated from male infertility or assisted reproduction rather than derived from RPL-specific trials. The amended AUA/ASRM guideline explicitly notes the absence of well-controlled studies showing that SDF-directed interventions reduce RPL, while RCOG similarly identifies the lack of prospective therapeutic evidence as a central limitation [2,14,15]. Varicocelectomy can reduce SDF in infertile men with clinical varicocele, but this has not been shown to reduce miscarriage or improve live birth in RPL [50]. Small antioxidant studies have demonstrated biomarker improvement without adequately powered reproductive outcomes, and the large SUMMER trial did not show improved ongoing pregnancy or reduced first-trimester pregnancy loss in a broader infertility population [52,54].

Further work is also required to distinguish direct RPL evidence from evidence borrowed from infertility and assisted reproduction. Repeated implantation failure, fertilisation failure, impaired embryo development, and general infertility are clinically adjacent but biologically and methodologically distinct from recurrent miscarriage. Findings from these populations may support mechanistic plausibility, but they should not be treated as equivalent evidence for RPL diagnosis or management.

Future research should prioritise multicentre prospective cohorts with standardised RPL definitions, detailed couple-level phenotyping, uniform test methodology, and follow-up through the subsequent pregnancy. Live birth should be the principal outcome, with miscarriage, time to conception, gestational age at loss, and adverse effects reported as core secondary outcomes. Randomised trials should determine whether management directed by a paternal test improves reproductive outcomes compared with standard RPL care. Cost-effectiveness, patient acceptability, access to reproductive urology, and the psychological consequences of identifying a paternal abnormality also require evaluation before expanded testing can be recommended.

## 7. Conclusions

Paternal factors contribute to the biological and clinical heterogeneity of recurrent pregnancy loss, but the strength and clinical relevance of the evidence differ substantially between domains. Paternal age, reproductive and medical history, body weight, lifestyle, medication use, and relevant exposures can be assessed with minimal burden and should form part of the initial couple evaluation. Focused male examination and conventional semen analysis are appropriate when infertility or suspected male reproductive disease is present.

SDF is the most extensively investigated advanced paternal test. Meta-analyses and several case–control studies report higher SDF in RPL, but contemporary findings are inconsistent, and prospective prediction of subsequent live birth remains unproven [6,8,10,30,38]. Its use is therefore best restricted to selected cases of unexplained RPL, particularly when concurrent infertility or reproductive urology assessment provides an additional clinical indication. Parental karyotyping should remain risk stratified, while sperm aneuploidy testing, oxidative stress assays, microbiome profiling, epigenetic testing, and proteomic approaches remain investigational.

The major unresolved issue is not whether paternal abnormalities can be detected, but whether their detection improves prognosis or changes management in a way that increases live birth. At present, no paternal biomarker has a sufficiently validated treatment pathway to justify routine biomarker-directed intervention. Male-directed treatment should therefore address established andrological disease and modifiable health factors rather than an isolated abnormal laboratory result.

Progress in this field requires a shift from association studies toward prospective prognostic validation and RPL-specific test-guided trials. Until such evidence is available, paternal assessment should remain risk-stratified, evidence-informed, and integrated within evaluation of the couple rather than applied as a separate or indiscriminate testing pathway.

## Figures and Tables

**Table 1:** Comparison of current guideline recommendations for paternal assessment in recurrent pregnancy loss.

Guideline	Clinical Paternal Assessment	Semen Analysis	SDF Testing	Genetic Testing	Male Referral or Treatment
**ESHRE 2023 [1]**	Assess paternal age and lifestyle factors	No specific recommendation	May be considered for diagnostic purposes	Parental karyotyping after individual risk assessment	No specific SDF-directed treatment pathway established
Recommendation grade/strength	Strong	Not graded	Conditional	Conditional	GPP for lifestyle Conditional for PICSI/antioxidants
Evidence level/quality	Low	Not graded	Moderate	Low	Not formally graded for lifestyle GPP; Very Low for PICSI/antioxidants
**ASRM 2026 [17]**	Review male history and relevant risk factors	Standard parameters are not predictive of RPL; perform if concurrent infertility is present	Recommended in selected cases of unexplained RPL or concurrent infertility	Parental karyotyping according to clinical context	Consider reproductive urology evaluation in selected cases
Recommendation grade/strength	Not formally graded	Not formally graded	Not formally graded; recommended in certain circumstances	Not formally graded; recommended in certain circumstances	Not formally graded; possible benefit in selected cases
Evidence level/quality	Observational evidence	Limited evidence; no demonstrated predictive value	Meta-analytic association evidence; treatment utility unproven	Established genetic/counselling rationale; no formal certainty grade	Limited/conflicting evidence; no controlled RPL-specific treatment evidence
**RCOG 2023 [2]**	Advancing paternal age recognised as a risk factor	No specific recommendation	Not routinely recommended outside research	Selective parental karyotyping	No routine male-directed treatment recommended
Recommendation grade/strength	Grade B	Not specifically graded	Grade D	Grade D/GPP depending on indication	Grade D
Evidence level/quality	2++	2+; inconsistent observational evidence	Level 4	Level 3/4, respectively	Level 3
**RANZCOG 2025 [18]**	Include paternal history and modifiable risk factors	Not specifically recommended as part of RPL screening	May be considered case by case for diagnostic purposes	Selective parental karyotyping	Specialist referral when clinically indicated
Recommendation grade/strength	Not separately graded	Not graded	Consider case-by-case	Conditional	Not separately graded
Evidence level/quality	Not separately graded	Not graded	Low	Not separately graded for parental karyotyping	Limited RPL-specific treatment evidence
**HSE/RCPI 2025 [19]**	Assess paternal history, age, lifestyle, medications, and body weight	No specific recommendation	Not routinely recommended outside research	Selective parental karyotyping	No evidence to recommend treatments for male factors
Recommendation grade/strength	2C (weak recommendation)	Not graded	2C (weak recommendation)	2C (weak recommendation)	2C (weak recommendation)
Evidence level/quality	Low	Not graded	Low	Low	Low
**SOGC 2025 [20]**	Assess age, BMI, lifestyle, heat or toxin exposure, infection, and varicocele	Not addressed	May be offered when no other abnormality has been identified	Selective parental karyotyping	Referral according to identified male factors; treatment evidence remains limited
Recommendation grade/strength	Not separately graded	Not addressed	Not stated	Conditional/Strong depending on indication	Not separately graded
Evidence level/quality	Not separately graded	Not graded	Not stated	Low/High depending on indication	Limited evidence
**DGGG/OEGGG/SGGG 2022 [3]**	Addresses paternal age and couple-level genetic risk	Not addressed	Not addressed	Parental karyotyping in selected couples	Not addressed
Recommendation grade/strength	No specific consensus recommendation	Not addressed	Not addressed	Expert consensus	Not addressed
Evidence level/quality	Paternal-age association reported; no formal evidence grade	Not graded	Not graded	Consensus level +++; no formal evidence-quality grade	Not graded
**AUA/ASRM Male Infertility 2024 [14,15]**	Evaluate the male partner in couples with RPL	Standard component of male infertility evaluation (not RPL-specific)	Evaluate men from RPL couples with SDF testing	Paternal karyotyping in RPL couples, additional male genetic testing according to infertility phenotype	Reproductive urology evaluation and treatment of established male disease
Recommendation grade/strength	Expert Opinion	Strong Recommendation	Moderate Recommendation	Expert Opinion	Expert Opinion for male reproductive evaluation
Evidence level/quality	Not formally graded	Grade B (general male infertility evaluation, not RPL-specific)	Grade C	Not formally graded	Insufficient RPL-specific treatment evidence
**EAU 2026 [16]**	Comprehensive male reproductive assessment	Perform when male infertility is suspected	Perform in RPL, unexplained infertility, or ART failure	Genetic testing according to male infertility indications	Treat established male pathology according to andrology indications; testicular sperm use for high SDF remains experimental
Recommendation grade/strength	Strong	Strong	Strong	Strong when indicated by male-infertility phenotype	Weak for selected varicocelectomy; Weak/experimental for testicular sperm
Evidence level/quality	LE 2a (general male-infertility evaluation)	LE 3; overall male evaluation LE 2a (not RPL-specific)	LE 2a	LE 2a	LE 2a/LE 3, respectively

**Note:** Recommendation grades and evidence levels are reported using each organization’s original grading system and are not directly comparable across guidelines. Where no domain-specific formal grading was provided, this is indicated as “not graded” or “not specifically graded,” with the evidence basis described where available. For RCOG, 2++ denotes high-quality observational evidence with a very low risk of bias or confounding, whereas 2+ denotes well-conducted observational evidence with a low risk of bias or confounding. For DGGG/OEGGG/SGGG, +/++/+++ indicate increasing levels of expert consensus. **Abbreviations:** ART, assisted reproductive technology; ASRM, American Society for Reproductive Medicine; AUA, American Urological Association; BMI, body mass index; DGGG, German Society of Gynaecology and Obstetrics; EAU, European Association of Urology; ESHRE, European Society of Human Reproduction and Embryology; GPP, Good Practice Point; HSE, Health Service Executive; OEGGG, Austrian Society of Gynaecology and Obstetrics; PICSI, Physiological Intracytoplasmic Sperm Injection; RANZCOG, Royal Australian and New Zealand College of Obstetricians and Gynaecologists; RCPI, Royal College of Physicians of Ireland; RCOG, Royal College of Obstetricians and Gynaecologists; RPL, recurrent pregnancy loss; SDF, sperm DNA fragmentation; SGGG, Swiss Society of Gynaecology and Obstetrics; SOGC, Society of Obstetricians and Gynaecologists of Canada.

**Table 2:** Evidence summary and clinical status of paternal assessment domains in recurrent pregnancy loss.

Domain	Evidence Base	Main Finding	Prognostic or Treatment Utility	Clinical Status
Paternal age	Meta-analysis and prospective cohorts	Miscarriage risk increases modestly with advanced paternal age, particularly after 40 years [4].	Limited value for predicting subsequent live birth [8]	Routine history and counselling
Obesity and metabolic health	Observational cohorts	Metabolic burden is associated with pregnancy loss, and paternal obesity with lower live-birth probability [5,8]	No RPL-specific evidence that intervention improves live birth	Routine clinical assessment
Conventional semen analysis	Small heterogeneous studies and meta-analyses	Differences in motility and morphology are inconsistent, while concentration is often similar between groups [28,29,30]	No validated prognostic value for subsequent miscarriage or live birth	Selective, mainly with concurrent infertility or suspected male disease
Sperm DNA fragmentation	Meta-analyses, case–control studies, and limited prospective data	SDF is frequently higher in RPL, but results vary by assay and population [6,10,11]	Subsequent live-birth prediction and benefit of SDF-directed treatment remain unproven [8]	Selective
Parental karyotyping	Systematic reviews and cohorts	Structural chromosomal abnormalities are detected in a minority of couples, with lower yield in unselected populations [46,47].	Clinically relevant when an abnormality is identified, but intervention evidence is limited	Selective and risk-stratified
Sperm aneuploidy testing	Meta-analysis and small case–control studies	Higher sperm aneuploidy has been reported in some RPL cohorts [7]	No validated prediction of subsequent outcome and no proven test-guided treatment	Investigational
Varicocele and oxidative stress	Mainly infertility evidence for varicocele and limited direct RPL evidence for oxidative stress	Varicocelectomy may reduce SDF in infertile men with clinical varicocele, while direct RPL data have not shown consistently increased seminal oxidative stress [39,50]	No direct evidence of reduced miscarriage or improved live birth in RPL	Treat varicocele only for established andrological indications; oxidative stress testing remains investigational
Antioxidant therapy	Small RPL subgroup studies and indirect infertility trials	Within-group biomarker improvements have been reported, but clinical benefit is unconfirmed [52]	No established improvement in pregnancy or live-birth outcomes [54]	Not recommended as routine biomarker-directed treatment
Infection and seminal microbiome	Limited observational and indirect evidence	Seminal HPV has been associated with miscarriage in mixed populations, but no reproducible RPL-specific microbiome signature has been identified [13,55]	No validated prognostic or antimicrobial treatment pathway	Investigational
Sperm epigenetics and other omics	Small discovery and diagnostic-development studies	Differential methylation and candidate biomarkers have been reported [9,12,56]	No independent prospective validation or treatment utility	Investigational

**Abbreviations:** HPV, human papillomavirus; RPL, recurrent pregnancy loss; SDF, sperm DNA fragmentation.

## Data Availability

No new data were created or analyzed in this study. Data sharing is not applicable to this article.
